# With the Permission of Microtubules: An Updated Overview on Microtubule Function During Axon Pathfinding

**DOI:** 10.3389/fnmol.2021.759404

**Published:** 2021-12-02

**Authors:** Carlos Sánchez-Huertas, Eloísa Herrera

**Affiliations:** Instituto de Neurociencias, Consejo Superior de Investigaciones Científicas-Universidad Miguel Hernández (CSIC-UMH), Alicante, Spain

**Keywords:** microtubules, microtubule-associate proteins, growth cone, neuronal cytoskeleton, axon guidance and pathfinding, +TIP

## Abstract

During the establishment of neural circuitry axons often need to cover long distances to reach remote targets. The stereotyped navigation of these axons defines the connectivity between brain regions and cellular subtypes. This chemotrophic guidance process mostly relies on the spatio-temporal expression patterns of extracellular proteins and the selective expression of their receptors in projection neurons. Axon guidance is stimulated by guidance proteins and implemented by neuronal traction forces at the growth cones, which engage local cytoskeleton regulators and cell adhesion proteins. Different layers of guidance signaling regulation, such as the cleavage and processing of receptors, the expression of co-receptors and a wide variety of intracellular cascades downstream of receptors activation, have been progressively unveiled. Also, in the last decades, the regulation of microtubule (MT) assembly, stability and interactions with the submembranous actin network in the growth cone have emerged as crucial effector mechanisms in axon pathfinding. In this review, we will delve into the intracellular signaling cascades downstream of guidance receptors that converge on the MT cytoskeleton of the growing axon. In particular, we will focus on the microtubule-associated proteins (MAPs) network responsible of MT dynamics in the axon and growth cone. Complementarily, we will discuss new evidences that connect defects in MT scaffold proteins, MAPs or MT-based motors and axon misrouting during brain development.

## Introduction

The navigation of neural axons to find appropriate synaptic partners is one of the most extraordinary events that take place during the development of the nervous system. Axon extension is led by an amoeboid-like cytoplasmic enlargement at the tip, denominated the growth cone (GC). This is a small but extremely dynamic and sensitive cellular structure that integrates extracellular guidance information and transduces the mechanical forces necessary for the steering and propulsion movements during axonal navigation. At the leading edge, the growth cone is composed of motile sheet-like lamellipodia and narrow filopodia that are sensitive to external guidance cues or ligands because they express specific receptors at the surface. Ligand-receptor signaling activates intracellular transduction pathways that primarily converge over growth cone cytoskeleton remodeling, which in coordination with substrate adhesions turnover and membrane trafficking, orchestrates the steering movements of the axon (Geraldo and Gordon-Weeks, 2009; Lowery and Vactor, 2009; Vitriol and Zheng, 2012; Kerstein et al., 2015).

The highly conserved collection of axon guidance proteins consists of attractive/repulsive membrane-anchored and secreted molecules. Five large families of canonical guidance proteins have been identified: netrins that signal through the deleted in colorectal cancer (DCC), Neogenin and UNC-5 receptors; Slits, that bind to their roundabout (Robo) receptors; Semaphorins, that activate both Neuropilin and Plexin receptors; Ephrins and Ephs; and Repulsive Guidance Molecule family (RGMs) that bind to Neogenin. Besides these initially identified families of guidance proteins, cell-adhesion molecules, growth factors and morphogens, such as the Wnts, Sonic hedgehog (Shh), TGF-β/BMP, neurotrophins or endocannabinoids have been implicated in axonal navigation (Kolodkin and Tessier-Lavigne, 2011; Yam and Charron, 2013; Zhou et al., 2014; Short et al., 2021). These guidance ligand-receptor modules have been identified and reported as essential for the formation of the commissural tracts in the spinal cord and forebrain, the retinotopic maps, the thalamocortical connections or the sensory motor innervation of the limbs, among other systems (Chedotal and Richards, 2010; Leyva-Díaz and López-Bendito, 2013; Chédotal, 2019; Herrera et al., 2019a).

During pathfinding GCs are simultaneously exposed to various signaling proteins and the final guidance decision relies on the spatial-temporal repertoire of guidance receptors expressed at the surface and the computation of their downstream signaling pathways. In addition, neuron-intrinsic molecular mechanisms including response-modulating co-receptors, receptor-receptor interactions, receptor clustering and oligomerization, proteolytic processing of receptors, or the trafficking of signaling receptors-carrying endosomes, contribute to the diversification of axonal responses to a same guidance cue (Dudanova and Klein, 2013; Pasterkamp and Burk, 2021; Zang et al., 2021). These intracellular pathways ultimately converge on proteins managing the cytoskeleton remodeling in the axon and GC.

The major networks constituting the mature neuronal cytoskeleton are formed by microtubules (MTs), actin fibers (F-actin) and neurofilaments, but axon pathfinding is mainly governed by F-actin and MTs acting coordinately in response to extracellular guidance signaling (Geraldo and Gordon-Weeks, 2009; Lowery and Vactor, 2009; Dent et al., 2011; Coles and Bradke, 2015). Seminal studies on the effects of F-actin disrupting drugs over invertebrate neurons in culture, revealed the critical role of the actin cytoskeleton in filopodia maintenance, GC turning and axonal pathfinding (Bentley and Toroian-Raymond, 1986; Zheng et al., 1996). Thereafter, an intricate network of actin-binding and regulatory proteins mediating the axonal response to guidance molecules has been progressively disclosed [see Table 1 in Dent et al. (2011) and Kolodkin and Pasterkamp (2013) for detailed information on actin-binding proteins steering the GC]. Most axon guidance pathways engage the Rho family of small GTPases, mainly represented by RhoA, Rac1 and Cdc42, via their activating guanine nucleotide exchange factors (RhoGEFs) and deactivating GTPase activating proteins (RhoGAPs) (Hall and Lalli, 2010). RhoGTPases, in turn, drive the activity of F-actin regulators, such as the nucleating Arp2/3 complex (Strasser et al., 2004; Shakir et al., 2008), the WASP nucleation factors (Shekarabi et al., 2005; Shakir et al., 2008), the polymerization regulators formins or the Ena/VASP family (Goode and Eck, 2007), the molecular motor Myosin II (Amano et al., 1998; Medeiros et al., 2006) or the severing protein ADF/cofilin (Kuhn et al., 2000), to modulate the actin-based filopodia and lamellipodia dynamics. The essential contribution of actin dynamics to axon guidance signal transduction in the GC has been reviewed recently by other authors (Omotade et al., 2017; Niftullayev and Lamarche-Vane, 2019) and, therefore, will not be the main focus of this review. For years, the long-standing view in the field was that the GC turns as a result of the stabilization/destabilization balance of the actin-rich filopodia and lamellipodia in the presence of a guidance cue and the MT cytoskeleton just provided structural support via MT-dependent transport to consolidate the actin-dependent turning events. However, the role of MTs in cellular functioning is continuously expanding and accumulating evidence indicate that MTs are not just passive regulators of GC dynamics. Instead, MTs can actively control GC protrusion and steering and, along with MT-associated proteins (MAPs), are direct targets of axon guidance signaling pathways (Gordon-Weeks, 2004; Dent et al., 2011; Kalil et al., 2011; Liu and Dwyer, 2014; Bearce et al., 2015; Cammarata et al., 2016; Kahn and Baas, 2016).

In the first part of this review we provide an overview on the configurations of the MT cytoskeleton in the axon and growth cone and describe the cytoskeletal mechanisms that drive GC directional responses, focusing on the contribution of MTs. In the second half of this article, we highlight recent evidences suggesting that guidance cues directly control the activity and localization of MT-associated proteins (MAPs) and discuss how alterations in genes encoding MT network regulators may cause an abnormal development of neural networks *in vivo*.

## The Neuronal Microtubule Cytoskeleton

Microtubules are hollow cylindrical structures composed of 13 laterally-associated protofilaments of α-tubulin and β-tubulin heterodimers assembled in a head-to-tail manner, conferring an intrinsic polarity characterized by a stable/slow-growing “minus-end” and a dynamic/fast-growing “plus-end” (Desai and Mitchison, 1997). In eukaryotic cells, the MT nucleation – the *de novo* MT formation from its minus end – is initiated by the γ-tubulin ring complex (γTuRC) in cooperation with additional proteins that regulate MT-nucleation kinetics. MT nucleation events are spatially restricted to MT-organizing centers (MTOC), which concentrate the γ-TuRCs, and the centrosome is a major MTOC in animal cells (Paz and Lüders, 2018). MT minus-ends are stabilized by a γTuRC cap (Wiese and Zheng, 2000) or by calmodulin-regulated spectrin-associated proteins (CAMSAPs) (Jiang et al., 2014). Instead, the MT plus-ends are more dynamic, alternating polymerization and shrinkage phases (catastrophes), a property commonly referred to as “dynamic instability” (Walker et al., 1989; Tran et al., 1997; Zhang et al., 2015). These MT-intrinsic dynamic plus-end transitions between growth and shrinkage can be externally regulated by the activity of other MAPs, that control the supply of soluble tubulin-heterodimers, the speed and duration of the polymerization/depolymerization events and the plus-ends resilience to collapse (reviewed in Akhmanova and Steinmetz, 2015).

At the onset of neuron differentiation, MTs nucleation takes place mainly at the centrosome. To meet axon growth needs, MTs are subsequently released from the centrosome, sorted into the axon and anterogradely transported by means of molecular motor forces (Kapitein and Hoogenraad, 2015). During maturation the neuron centrosome progressively loses its MT-nucleating and MT-organizing skills to such an extent that axonal MT growth, axonal extension or overall neural development do not require a centrosome (Basto et al., 2006; Stiess et al., 2010; Nguyen et al., 2011). Indeed, during the last years centrosome-independent MT nucleation activities has been identified within the axon and dendrites of mammalian and invertebrate neurons (Stiess et al., 2010; Ori-McKenney et al., 2012; Nguyen et al., 2014; Sánchez-Huertas et al., 2016; Cunha-Ferreira et al., 2018; Qu et al., 2019; Liang et al., 2020). MT configurations display differently in axons and dendrites. In mammalian neurons, MTs are uniformly oriented in the axons, with their plus-ends toward the tip, while in dendrites MTs are arranged with mixed polarity. The uniform MT plus-end-out polarity of axons is mainly established and maintained by motor-dependent MT sliding mechanisms (Miller and Suter, 2018) and the spatial-temporal control of non-centrosomal MT nucleation (Wilkes and Moore, 2020). For example, the cytoplasmic dynein motor promotes bulk forward translocation of MTs into the axon and clears the minus-end-out MTs from axons by soma-directed sliding (Roossien et al., 2014; Rao et al., 2017). In addition, new plus-end-out MTs are locally generated from the lateral surface of pre-existing axonal MTs by the Augmin-γTuRC module (Sánchez-Huertas et al., 2016; Cunha-Ferreira et al., 2018) and TRIM46 bundles these plus-end-out MTs (van Beuningen et al., 2015), to strengthen the axonal identity.

Neuronal MTs show specific physical and dynamic features enabled by their differential tubulin isotype composition, assorted post-translational tubulin modifications – including tyrosination, acetylation or polyglutamylation – and a neuron-specific MAP network unevenly distributed over the axonal and somatodendritic compartments (Moutin et al., 2021). Within the axon shaft, MTs are heavily stabilized and organized in dense, parallel and overlapping bundles. This longitudinally aligned MT network enable the directional transport of organelles, vesicles, other MTs and cargoes along the axon, mediated by MT-based motor proteins of the kinesin superfamily and cytoplasmic dynein (Hirokawa et al., 2010; Leterrier et al., 2017). By virtue of the differential MT layouts in neuron compartments, uniform in axons and mixed in dendrites, specific motor-driven cargos are selectively sorted, determining axonal specification, maturation and navigation (Guillaud et al., 2020).

## Microtubule Cytoskeleton in the Growth Cone

**FIGURE 1:**
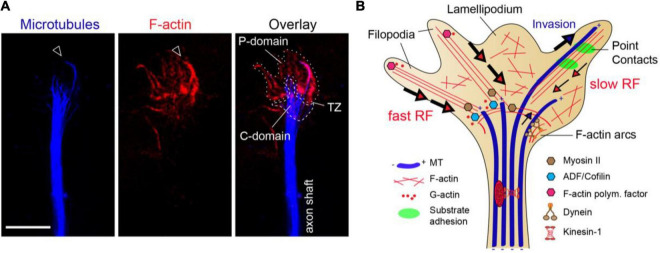
The growth cone cytoskeleton. **(A)** High-resolution image of an axonal growth cone labeled with phalloidin (red) and α-tubulin (blue) from a hippocampal neuron culture. Actin filaments (F-actin) and microtubules (MTs) are segregated amid the peripheral (P-domain) and central regions (C-domain), respectively. Arrowhead marks isolated MTs invading the P-domain aligned with F-actin bundles. P-domain and C-domain are outlined using a dotted or dashed lines, respectively. TZ, transition zone. Scale bar, 5 μm. **(B)** The clutch model for growth cone protrusion and steering. F-actin (red lines), microtubules (blue thick tubes), MT plus-ends (+), point contacts of adhesion (green). Filopodia is formed by F-actin bundles and lamellipodia by F-actin meshworks. The F-actin retrograde flow at the P-domain is balanced between F-actin polymerization and depolymerization rates, and myosin-based pulling forces at the TZ. Actomyosin forces at the TZ restrain MT entry into the P-domain. The engagement of F-actin to adhesion point contacts slows the F-actin retrograde flow rate and facilitates MT invasion to the growth cone periphery, determining outgrowth and steering.

At the axon tip, the growth cone (GC) can be subdivided in several areas: a mobile peripheral (P) domain, containing filopodia and lamellipodia, a central (C) domain and a transition zone (TZ) in between. The cytoskeletal networks are organized in a highly segregated fashion among these GC subdomains (Figure 1A). The P-domain is mainly made of actin fibers organized in dense bundles or loose F-actin meshworks, originating the filopodia and lamellipodia, respectively. The C-domain is populated by thickly bundled MTs, which are continuously pushed by actin-based Myosin II-dependent rearwards forces working at the TZ. Only isolated MTs can pass this actomyosin-mediated barrier to MT assembly and invade the actin-rich P-domain, where MTs display “dynamic instability” (Figures 1A,B) (Letourneau, 1983; Forscher and Smith, 1988; Dent and Kalil, 2001; Schaefer et al., 2002; Zhou et al., 2002). F-actin bundles at the GC P-domain experience a sustained retrograde flow (RF) product of: (i) the continuous F-actin polymerization at the submembranous cortex, (ii) the retrograde Myosin-dependent pulling forces and (iii) the F-actin depolymerizing activity of ADF/Cofilin at the transition zone. Actomyosin arcs contribute to MT bundling and advance into the C-domain by exerting forces along the side of the neck of the GC (Figure 1B) (Medeiros et al., 2006; Burnette et al., 2008). In addition, once entering the P-domain, MTs tend to unfasciculate, bend and loop due to a dynamic MT-F-actin interplay enabled by MT-F-actin coupling proteins, including MAPs or MT plus-tip interacting proteins (+TIPs) (Coles and Bradke, 2015; Cammarata et al., 2016). This transient MT-F-actin coupling mechanism mediated by MAPs enables MT capture and guidance by F-actin bundles at the P-domain, but also exposes MTs to the continuous F-actin retrograde flow, influencing the orientation and speed of MT growth. This F-actin retrograde flow drags MTs backward and clears the GC periphery of MTs, by attenuating and buckling the MT trajectories. Opposing to these retrograde forces over MT dynamics, the cytoplasmic dynein motor exerts anterograde MT-sliding movements to introduce MTs in the P-domain (Figure 1B) (Forscher and Smith, 1988; Zhou et al., 2002; Suter et al., 2004; Myers et al., 2006; Grabham et al., 2007; Schaefer et al., 2008; Marx et al., 2013). Therefore, MT distribution in the GC is partially determined by F-actin network at the same time that the F-actin-based filopodia and lamellipodia dynamics is also known to be strongly influenced by MT capture and stabilization at the GC periphery (Sabry et al., 1991; Tanaka et al., 1995; Gallo, 1998). Such intense reciprocal regulation between MT and F-actin networks in the GC highlights the importance of an expanding family of MT-actin crosslinking proteins in axon guidance decisions, but the precise nature of MT-actin interlinking mechanisms remain to be elucidated.

Traction forces that propel axon outgrowth rely on clutching forces exerted at substrate adhesion points, which generally assemble within growth cone filopodia. At these point contact adhesions, extracellular matrix (ECM) proteins activate integrin receptors that recruit scaffolding and signaling proteins, which physically link to the F-actin cytoskeleton (Suter et al., 1998; Woo and Gomez, 2006; Bard et al., 2008; Shimada et al., 2008; Myers and Gomez, 2011; Toriyama et al., 2013). This molecular “clutch” restrains myosin-II mediated F-actin contractile forces and increases the pushing forces of actin polymerization toward the leading edge membrane, producing GC protrusion. Then, taking advantage of the attenuated F-actin retrograde flow, pioneer MTs invade the filopodia and eventually get captured and stabilized. The stabilized MTs enable the entry of organelles and vesicles to the GC periphery, powered by MT-based motor forces, and the GC stepwise progresses toward the engorgement and consolidation stages (Figure 1B) (Suter et al., 1998; Zhou et al., 2002; Gomez and Letourneau, 2014; Kerstein et al., 2015). This is the currently accepted mechanistic model for axon outgrowth, although recent data has revealed some inconsistencies (Santos et al., 2020; Turney et al., 2020). By analyzing GC protrusion and axon growth over three-dimensional (3D) matrices, Santos et al. (2020) showed that actomyosin forces do not restrain MTs at the C-domain of GCs in this environment. Instead, MTs widely populate the P-domain of the GCs, enabling a rapid axon elongation. In addition, the authors showed that axons can polarize and extend in adhesion-inert 3D matrices, suggesting that cell adhesions may be dispensable for axon growth in a 3D environment (Santos et al., 2020). GC motility and axon advance has also been analyzed over non-adhesive substrate gaps in a 2D environment. This study revealed that axons transiently stop at gaps, but GC protrusive activity continued, with MTs entering the filopodium extending across the gap. These MTs were powering the necessary molecular forces for GC to pass over the non-adhesive substrate (Turney et al., 2020). Experimental evidence has extensively validated the clutch hypothesis during *in vivo* axon wiring (Berezin and Walmod, 2014), so this new data suggests the existence of additional regulatory levels in the mechanism of GC motility.

A classic study analyzing the *in vivo* GC morphology of retinal axons performed in the 80’s described that retinal axons display long and slender GCs when navigating the optic nerve, while at the guidance decision point of the optic chiasm GCs become shorter, wider and grow multiple filopodia (Bovolenta and Mason, 1987). The study revealed that GC behaviors, and the underlying mechanisms, can differ between bulk navigation regions and guidance decision environments. This sets a model out where GCs may either waive or activate substrate adhesion mechanisms according to extracellular guidance factors or neuron-intrinsic commands (Padmanabhan and Goodhill, 2018), integrating the *a priori* antagonistic evidences previously exposed. However, further work is needed to fully understand the mechanotransduction events during *in vivo* axon navigation.

### Microtubules as the Mechanistic Effectors of Growth Cone Turning

The reorganization of the GC cytoskeleton during axon steering is ultimately enabled by a tight regulation of the MT-actin interplay (Gordon-Weeks, 2004; Lowery and Vactor, 2009; Dent et al., 2011). The targeting of the actin cytoskeleton, either by downregulation of actin isoforms or by depolymerization of F-actin using Cytochalasin B, reduces the size of the growth cone, hinders filopodial dynamics and abolishes the axonal turning response (Forscher and Smith, 1988; Zheng et al., 1996; Moradi et al., 2017). Despite the requirement of F-actin for axon steering, it has been shown that neurons can still elongate their axons after F-actin depolymerization or inhibition of myosin II (Bentley and Toroian-Raymond, 1986; Zheng et al., 1996; Bradke and Dotti, 1999; Hur et al., 2011b), indicating that F-actin filaments remodeling is essential for motility and steering but dispensable for axon extension. In contrast, MT dynamics is necessary for axon growth (Yamada et al., 1970; Bamburg et al., 1986; Mansfield and Gordon-Weeks, 1991; Tanaka et al., 1995; Yu and Baas, 1995; Rochlin et al., 1996; Tischfield et al., 2010) but the role of MTs in GC steering is yet a matter of discussion.

More than 30 years ago, pioneer studies already showed that MTs asymmetrically invade the P-domain of the GC in the direction of the turn and their reorganization is essential during GC maneuvers (Forscher and Smith, 1988; Sabry et al., 1991; Tanaka et al., 1995; Williamson et al., 1996). In addition, MTs entering the filopodia were found to be captured and stabilized by preventing shrinkage. These stabilized MTs allowed the flow of cytoplasmic organelles and increased the filopodia lifetimes, enabling directional axon outgrowth (Gordon-Weeks, 1991; Sabry et al., 1991; Geraldo et al., 2008). Ever since, different works have demonstrated that axon guidance signaling proteins influence MT dynamics at the GCs. For instance, bath incubation of Sema3A and netrin-1 in neuron cultures revealed that Sema3A treatments result in the collapse of MT networks, while netrin-1 incubation stimulates MT splaying and exploration in the GC peripheral region (Dent et al., 2004; Shao et al., 2017). Nerve growth factor (NGF) signaling in sensory neurons also redistributed the MTs in the distal part of axons (Zhou et al., 2004; Turney et al., 2016), and Wnt3a or Wnt5a treatments changed the organization and directionality of MT polymerization in the GC (Purro et al., 2008; Li et al., 2014). Micro-gradients of Brain-derived neurotrophic factor (BDNF) or Sema3A over GCs of sensory axons also biased the direction of MT growth toward the treated GC side or the opposite side, respectively (Pavez et al., 2019). In addition, genetic studies in *Caenorhabditis elegans* showed that UNC-6/Netrin signaling hampers MT accumulation in the GC and inhibits protrusion, through a molecular pathway that involves the repulsive Netrin receptor UNC-5, RHO-1/RhoA and UNC-33/CRPM (Gujar et al., 2019). Consistently with these findings, the inhibition of MT dynamics blunts GC turning in response to guidance cues. The exposure of GCs and axons to drugs that disrupt MT dynamics, such as taxol or nocodazole, reduced the GC activity and abolished the turning of GCs exposed to netrin-1 and glutamate gradients or substrate boundaries (Tanaka and Kirschner, 1995; Challacombe et al., 1997; Buck and Zheng, 2002; Suter et al., 2004). Yet, because the spatial distribution of MTs in the GC periphery is strongly influenced by actomyosin forces and F-actin dynamics, MTs have been classically relegated to simple supporters of actin-guided movements during axon navigation.

Now, mounting evidences support the idea that MTs and MAPs can also instruct axon pathfinding even when actin dynamics are not directly perturbed. Seminal studies performed 20 years-ago showed that the focal application of the MT stabilizing drug taxol to one side of the GC induces turning toward the drug source, whereas the application of the MT destabilizing drug nocodazole triggers GC turning away from the application side (Buck and Zheng, 2002). Experimental evidence also demonstrated that MT-initiated GC turning engages F-actin remodeling during the movement, since the inhibition of actin polymerization abolished the taxol-evoked attractive GC turning (Buck and Zheng, 2002). Subsequent works employing micro-scale chromophore-assisted laser inactivation (micro-CALI) have also revealed that several MAPs can trigger GC steering. Micro-CALI technique has been exploited to address the role of specific proteins in GC steering (Chang et al., 1995; Diefenbach et al., 2002), although it may have caveats, such as a rapid recovery dependent on protein diffusion or trafficking. The asymmetric inactivation of the MT-stabilizing protein MAP1B or the Adenomatous polyposis coli (APC) protein – a +TIP that stimulates MT polymerization – by micro-CALI in one side of the GC, led to the collapse of the irradiated side followed by GC turning toward the opposite direction (Mack et al., 2000; Koester et al., 2007). In contrast, the asymmetric inactivation of the MT extension-modulator CRMP2 or the MT-sliding motor Kinesin-5 in half of the GC by micro-CALI, resulted in GC turning toward the irradiated side (Higurashi et al., 2012; Nadar et al., 2012). Overall, these experiments support the idea that MT dynamics are not just required for guidance-evoked GC steering movements, but they play an instructive role in GC turning. Corroborating their instructive role in axon guidance signal transduction, evidences from Liu’s laboratory have proven that the neuron-specific tubulin isotype TUBB3 – polymerized in MTs – is a direct target of netrin-1 signaling (Qu et al., 2013; Huang et al., 2015; Shao et al., 2017). Netrin-1 is a dual guidance cue that can evoke attractive and repulsive responses by binding to its high-affinity GC receptors DCC and UNC5, respectively (Alcántara et al., 2000). It was reported that the exposure of cortical neuron cultures to netrin-1 induces GC chemoattraction and stimulates the MT dynamics in the GC. Both of these netrin-1 effects required the direct interaction of its receptor DCC with the neuron-specific tubulin isotype TUBB3, integrated in the MT polymer. Indeed, the interaction of TUBB3 with DCC was greatly increased by exogenous netrin-1 addition and this interaction was dependent on MT dynamics (Qu et al., 2013). Conversely, the GC repulsion induced by netrin-1 exposure through UNC5C receptor signaling also relied on TUBB3-UNC5C binding. UNC5C directly interacts with polymerized TUBB3 *in vitro* and both partially colocalize in the GC periphery of primary neurons. The focal application of Netrin-1 was found to disengage UNC5C-TUBB3 interaction in GCs and stimulate MT polymerization in the GC region distally to the netrin-1 source, promoting the repulsive response (Shao et al., 2017). Missense mutations of *TUBB3* in humans are associated to an abnormal development of the corpus callosum, the anterior commissure, the corticospinal tracts or optic nerves in human patients (Table 1). Additionally, it was found that *TUBB3* mutations impaired MT dynamics and abolished both netrin-1-evoked attractive and repulsive responses of cortical axons *in vitro* (Poirier et al., 2010; Tischfield et al., 2010; Whitman et al., 2016; Huang et al., 2018; Shao et al., 2019). Although the deficits caused by *TUBB3* loss-of-function in neural circuits development may be compensated by the remaining β-tubulin isotypes (Latremoliere et al., 2018).

**TABLE 1:** Links of microtubule-associated proteins (MAPs) with axon guidance.

Roles in MT networks	MAP	Function on GC motility	Axon tract development in animal models	Guidance pathways participated	Nerve tract associated pathology in humans	Actin crosstalk
Structural	TUBB3	Interaction with DCC or UNC5 increase/decrease upon netrin-1 signaling, interfering with MT dynamics and promoting both the attractive and repulsive responses of the GC.	Disease-associated *Tubb3* mutant mice show abnormal AC, CC and cranial nerves (Tischfield et al., 2010; Latremoliere et al., 2018)	Netrin-1-DCC (Qu et al., 2013); Netrin-1-UNC5 (Shao et al., 2017)	CFEOM3. Defects in the CC, AC or corticospinal tracts. Asymmetric cortical dysplasia and gyral disorganization (Poirier et al., 2010; Tischfield et al., 2010)	–
Structural	TUBA1A	Loss-of-function hindered neurite outgrowth in cortical neurons and altered GC cytoskeleton	*Tuba1a* ko mice show abnormal development of forebrain commissures (Buscaglia et al., 2020)	–	Abnormalities of the CC and basal ganglia/internal capsule. Lissencephaly and other cortical and cerebellar dysgenesis (Romaniello et al., 2018)	–
Structural	TUBB (TUBB5)	Altered MT dynamics and MT-based transport in patient’s fibroblasts	–	–	Hypoplasia or partial agenesis of the CC, and other cortical and cerebellar dysgenesis (Romaniello et al., 2018)	–
Nucleation modulator	TPX2	Localizes to neurite tips together with RanGTP to promote local MT nucleation in hippocampal neurons	–	–	–	–
Stability	MAP1B	Phospho-MAP1B stabilizes MTs at the GC periphery. Phosphorylated by GSK3β and CDK5 upon guidance signaling	*Map1b* ko mice display defective cortical and thalamocortical wiring, and CFEOM (Meixner et al., 2000; Del Río et al., 2004; Cheng et al., 2014)	Netrin-1 (Del Río et al., 2004); Draxin-DCC (Meli et al., 2015); Sema3A (Takabatake et al., 2020)	White matter deficit, hypoplasia of the CC (Walters et al., 2018)	Binds F-actin *in vitro*. Coordinates MTs and F-actin remodeling in DRG GCs (Villarroel-Campos and Gonzalez-Billault, 2014)
Stability	Tau	Hyperphosphorylated tau detaches from MTs and compromises MT stability in the GC. Phosphorylated by GSK3β, CDK5, or CaMKII upon guidance signaling	No phenotype in *tau* ko mice likely due to function overlapping with MAP1B	Sema3A (Sasaki et al., 2002); Wnt5a (Li et al., 2014; Biswas and Kalil, 2018); EphrinB1-EphB2 (Jiang et al., 2015); Sema3C (Moreno-Flores et al., 2004)	–	Crosslinks MT-F-actin *in vitro* (Elie et al., 2015). Couples MT and F-actin in GCs of cortical neurons (Biswas and Kalil, 2018)
Polymerization/stability	CRMP2	Non-phosphorylated CRMP2 transports tubulin heterodimers to distal axons via kinesin-1, to support MT growth. Phosphorylated sequentially by CDK5 and GSK3β upon guidance signaling	*Crmp2* ko mice display abnormal development of peripheral nerves and CC (Ziak et al., 2020)	Sema3A (Goshima et al., 1995); Sema4D-plexinB1 (Ito et al., 2006): RGMa (Wang et al., 2013); EphrinA5 (Arimura et al., 2005)	–	Binds the actin regulators: cytoskeleton a2-chimaerin and Sra-1/WAVE1 complex in axons (Brown et al., 2004; Kawano et al., 2005)
Stability	DCX	Stabilizes MT in the GC periphery. Phosphorylated by CDK5 upon Sema3A signaling, resulting in MT destabilization	*Dcx/Dclk1* ko mice show widespread defects in brain axon tracts (Deuel et al., 2006; Koizumi et al., 2006)	Netrin-1 (Fu et al., 2013); Sema3A (Bott et al., 2020)	Lissencephaly and double cortex syndrome (laminar heterotopias) (Bahi-Buisson et al., 2013)	Binds the actin-binding protein Spinophilin to organize F-actin. Coordinates MTs and F-actin in GCs (Tsukada et al., 2005; Tint et al., 2009)
Instability	SCG10	Active (non-phosphorylated) SCG10 destabilizes MTs, stimulating MT dynamics and promoting axon outgrowth and regeneration	–	EphB (Suh, 2004); Sema4D-PlexinB1? (Oinuma et al., 2004; Li Y.-H. et al., 2009)	–	–
Severing	Spastin	Spastin isoform M1 represses BMP guidance signaling during spinal motor axon pathfinding in developing zebrafish	–	BMP (Jardin et al., 2018)	Hereditary spastic paraplegia (Roll-Mecak and Vale, 2008)	–
Severing	Fignl1	Involved in spinal motor axons wiring during zebrafish development	–	–	–	–
Polymerization inhibition	KIF21A	Decreases MT polymerization and suppresses catastrophes, modulating the GC morphology, axon growth and pathfinding	Mutant *Kif21a* mice show defects in oculomotor nerves development (Cheng et al., 2014)	Sema3F (van der Vaart et al., 2013)	CFEOM1 (Yamada et al., 2003)	Binds and regulates the localization of Kank1, an F-actin polymerization inhibitor (Kakinuma and Kiyama, 2009)
Pausing	KIF21B	Accumulates in MT plus-ends and acts as autonomous pausing factor	*Kif21b* ko mice display thinner CC (Kannan et al., 2017)	–	Agenesis of the CC and microcephaly (Asselin et al., 2020)	Associates with ELMO1, a Rac1 regulator (Morikawa et al., 2018)
Polymerization inhibition	KIF2A	Prevents MT overstabilization in the GC.	*Kif2a* ko mice show aberrant overextension of hippocampal axons (Homma et al., 2003)	–	Malformations of cortical development, including microcephaly and gyration phenotypes (Poirier et al., 2013)	–
Transport	Dynein motor complex	Retrograde transport of signaling endosomes. Antiparallel MT sliding	–	NGF (Sainath and Gallo, 2015)	Polymicrogyria and Charcot-Marie-Tooth disease type2 (Poirier et al., 2013)	–
Transport	Kinesin-5	Antiparallel MT sliding. Blocks MT invasion into the GC periphery and determines GC turning. Required for evoked-turning response	–	–	Microcephaly and chorioretinopathy (Jones et al., 2014)	–
Transport	Kinesin-1 motor complex	Axonal transport of CB1R in hippocampal neurons	*Klc1* ko mice show pathfinding defects in corticofugal axons (Saez et al., 2020)	Endocannabinoids (Saez et al., 2020)	*Kif5C*: microcephaly, gyration phenotypes and white matter dysgenesis (Poirier et al., 2013; Michels et al., 2017)	–
Transport	KIF13B	Transports the F-actin-based motor Myosin X and its cargo DCC anterogradely along axons upon guidance signaling	–	Netrin-1-DCC (Yu et al., 2020)	–	–
Transport	KIF1BP	–	*Kif1bp* ko mice show defects in the anterior commissure and sympathetic innervation, but not in CC (Hirst et al., 2017)	–	Microcephaly, peripheral neuropathy (Goldberg-Shprintzen syndrome) (Drévillon et al., 2013)	–
Transport	KIF1Bβ	Axonal transport of IGF1R to mediate IGF-1-induced axon growth	*Kif1b* ko mice show abnormal development of the CC (Zhao et al., 2001)	IGF1-IGF1R (Xu et al., 2018)	Charcot-Marie-Tooth disease type 2A (Zhao et al., 2001)	–
Polymerization/scaffold	EB1, EB3	Guidance signaling instructs the asymmetric invasion of EB-labeled MT plus-ends or the MT polymerization dynamics	–	Sema4D-plexin (Laht et al., 2012, 2014); SDF1-CXCR4 *via* EB1/Drebrin module (Shan et al., 2021); BDNF, Sema3A *via* EB3/STIM1 module (Pavez et al., 2019)	–	EB3/drebrin coordinates MT-actin and regulates F-actin dynamics (Geraldo et al., 2008; Mizui et al., 2009; Mikati et al., 2013; Grintsevich and Reisler, 2014)
Stability	CLASP	Phosphorylation by Abl and GSK3β upon guidance signaling determines MT plus-end binding	–	Slit-Robo (Lee et al., 2004). PDGF (Engel et al., 2014).	–	Binds F-actin *in vitro* and regulates F-actin networks in sensory GCs (Marx et al., 2013)
Stability/RNA transport	APC	Asymmetric accumulation of APC in the GC anticipates the steering movement. Guidance signaling modulates APC MT plus-end binding via PI3K-GSK3β activity	*Apc* ko mice show widespread white matter defects (Yokota et al., 2009)	NGF (Zhou et al., 2004; Villarin et al., 2016); Wnt3a (Purro et al., 2008)	–	Regulates mDia and IQGAP1 (Watanabe et al., 2009; Okada et al., 2010). Required for MT-dependent F-actin assembly in hippocampal GCs (Efimova et al., 2020)
Stability	APC2	Defines the guidance of retinal ganglion cell axons at the chiasm midline	–	EphrinA2 (Shintani et al., 2009); Wnt5a (Morenilla-Palao et al., 2020)	–	Regulates actin dynamics through the formin DIA in *Drosophila* (Zhou et al., 2011)
Crosslink/arrangement	MACF1	Links MTs and F-actin. Coordinates MTs and F-actin interaction to organize the axonal cytoskeleton	Midline axon guidance in flies (Lee et al., 2007). *Macf1* ko mice show widespread white matter defects (Chen et al., 2006; Ka and Kim, 2016)	Wnt-β catenin (Chen et al., 2006)	Thin CC and AC, with lissencephaly (Dobyns et al., 2018)	Binds, stabilizes and organizes F-actin configurations (Kodama et al., 2003)
Stability/crosslink	NAV1	Stabilizes paused MT plus-ends. Couples MTs and F-actin in the GC of hippocampal neurons	–	Netrin-1 (Martínez-López et al., 2005; Sánchez-Huertas et al., 2020)	–	Binds F-actin *in vitro*, crosslinks MT-F-actin. Recruits the Trio to MT plus-ends (van Haren et al., 2014; Sánchez-Huertas et al., 2020)
Polymerization/nucleation modulator	XMAP215	Promotes MT entry in filopodia, regulates GC morphology and axon outgrowth in *Xenopus* neurons	–	EphrinA5 (Slater et al., 2019)	–	Co-aligns MTs and F-actin in GCs (Slater et al., 2019)
Polymerization	TACC3	Forms a complex with XMAP215. Phosphorylated by Abl. Phospho-mutants interfere with axon pathfinding	–	Slit2, EphrinA5 (Erdogan et al., 2017, 2020)	–	–
Crosslink	Gas2L1	Regulates axon outgrowth and branching	–	–	–	Stabilizes F-actin upon MT-F-actin interaction (Willige et al., 2019)
Stability/crosslink	DAAM	Actin assembly factor involved in axon growth and guidance. Regulates GC filopodia dynamics also *via* interaction with + TIPs	–	Wnt5 (Gombos et al., 2015)	–	Crosslinks MT and F-actin *in vitro* and coordinates the GC cytoskeleton in *Drosophila* neurons (Szikora et al., 2017)
Stability/crosslink	mDia1, mDia3	Actin assembly factor involved in axon growth and guidance. Binds and stabilizes MTs	Double *mDia* ko mice show midline crossing defects in the spinal cord (Toyoda et al., 2013)	EphrinA5, EphrinB3, Sema3A (Toyoda et al., 2013); SDF1-α (Arakawa et al., 2003)	–	Play dual roles in actin and MT dynamics (Thurston et al., 2012)
Stability/crosslink	FMN2	Enables MT capture by F-actin bundles and focal adhesion-based traction in filopodia	Fmn2 depletion impairs midline crossing in chick spinal cord (Sahasrabudhe et al., 2016)	Wnt (Lian et al., 2016)	–	Couples MTs and F-actin in GCs (Kundu et al., 2021)

*MT, microtubules; F-actin, actin fibers; GC, growth cone; +TIPS, MT plus-end interacting proteins; CFEOM, congenital fibrosis of the extraocular muscles; CC, corpus callosum; AC, anterior commissure; GC, growth cone; NGF, nerve growth factor; BDNF, brain-derived neurotrophic factor; BMP, bone morphogenetic protein; DRG, dorsal root ganglia.*

Other mutations in human α-and β-tubulin-encoding genes – such as *TUBA1A, TUBB2B, TUBA8, TUBB4A, TUBB2A, TUBB* – are linked to severe brain malformations and motor-cognitive disabilities, collectively refereed as tubulinopathies. These syndromes present gross brain malformations and an abnormal development of various nerve tracts, suggesting a putative role in axon guidance (Romaniello et al., 2018). Recent analysis performed on *TUBA1A* loss-of-function mice and cultured fibroblasts from *TUBB*-associated tubulinopathy patients have revealed an impaired MT dynamics and aberrant cytoskeleton configurations in the axonal GCs (Buscaglia et al., 2020; Sferra et al., 2020). However, it remains to be uncovered whether and which guidance molecules are involved in these MT-associated axon tract malformations.

## Microtubule-Associated Proteins in Axon Guidance

The assembly, stability and remodeling of MT networks during axon navigation mostly relies on the localization and activity of a wide range of MAPs located in the axon and GC compartments. MAPs manage many aspects of the MT cytoskeleton, including the spatial-temporal control of MT nucleation, polymerization, depolymerization, stability, pausing, bundling, severing, trafficking or interaction with other cellular structures (Goodson and Jonasson, 2018). Therefore, MAPs play a pivotal role in the transduction of attractive and repulsive guidance signaling over MT dynamics in axons and growth cones. Consistently, mutations in human MAP-coding genes have been associated to a wide spectrum of neurodevelopmental disorders linked to axon misrouting (Poirier et al., 2013; Chilton and Guthrie, 2017; Lasser et al., 2018; Romaniello et al., 2018). In this section we will summarize the intracellular pathways downstream axon guidance signaling that directly control the activity, localization or expression of MAPs to achieve GC protrusion and steering, as well as MAPs requirement for axon tract development *in vivo*. For clarity, we have classified the MAPs as: (1) MT-nucleation MAPs, (2) MT-stabilizing and polymerization-supporting MAPs, (3) MT-severing, destabilizing and polymerization-inhibitory MAPs, (4) MT-tracking motor proteins and (5) MT plus-tip interacting proteins (+TIPs).

### Microtubule-Nucleation Microtubule-Associated Proteins

In the shaft of cortical axons, MTs are formed *de novo* – nucleated – locally in an acentrosomal manner, by a mechanism involving the Augmin/HAUS complex and the γ-tubulin ring complex (γTuRC) that ensures the uniform polarity of the MT network (Sánchez-Huertas et al., 2016; Cunha-Ferreira et al., 2018). Although local events of MT nucleation have not been yet reported in the axonal GCs, acentrosomal γTuRC-dependent MT nucleation has been recently observed over endosomes in the dendritic GCs of invertebrate neurons (Liang et al., 2020; Yoong et al., 2020). These evidences, in combination with the following recent findings, allow to put forward the hypothesis of axon guidance signaling influencing local MT nucleation events in the distal axon.

The γTuRC-dependent MT nucleation in eukaryotic cells undergoes spatial and temporal regulation by means of additional MAPs, such as TPX2 and its activator RanGTP, and both proteins have been found to be enriched at neuritic tips (Chen et al., 2017; Huang et al., 2020; Liu et al., 2021). MT-bound TPX2 participates in MT nucleation at elongating neurite tips in cultured hippocampal neurons (Chen et al., 2017). RanGTP is transported anterogradely along the axons through actin waves, it colocalizes with actin-based structures in the axonal GC and enables local nucleation events at neurite tips (Chen et al., 2017; Huang et al., 2020). Actin waves (also known as growth cone-like waves) are dynamic cytoskeletal structures traveling anterogradely along the axon shaft. These waves are associated to transient MT generation activity along the axons, including an increase in MT polymerization and MT-based transport (Winans et al., 2016). Therefore, it is possible that RanGTP and TPX2 are transported to the GC, jointly with other MT nucleation machinery such as γ-TuRCs, to trigger local short-lived MT nucleation events.

New results also suggest that Wnt signaling could shape axonal MT configurations via regulation of local MT nucleation mechanisms. Weiner et al. (2020) showed that in *Drosophila*, some Wnt signaling proteins, such as Fz, LRP5/6 or Axin, recruit the MT core-nucleation protein γ-Tubulin to endosomes in the dendritic branch points, enabling local MT nucleation and indicating that extracellular Wnt signaling can regulate local MT nucleation in dendrites. In addition, two other recent studies have revealed that the Wnt pathway controls axon specification in developing neurons by organizing the polarity of MT networks both in the axon (Stanganello et al., 2019) and in non-axonal neurites (Puri et al., 2021). Plus, it is known that local MT nucleation contribute the MT arrangements in these compartments (Sánchez-Huertas et al., 2016; Cunha-Ferreira et al., 2018). Overall, these results convey a putative mechanism whereby extracellular Wnt signaling might control MT architecture in axons and dendrites via spatial-temporal control of MT nucleation in developing neurons. Hence, we believe that the contribution of local MT nucleation events in distal axons to guidance cue-instructed navigation should be further investigated.

### Microtubule-Stabilizing and Polymerization-Supporting Microtubule-Associated Proteins

**FIGURE 2:**
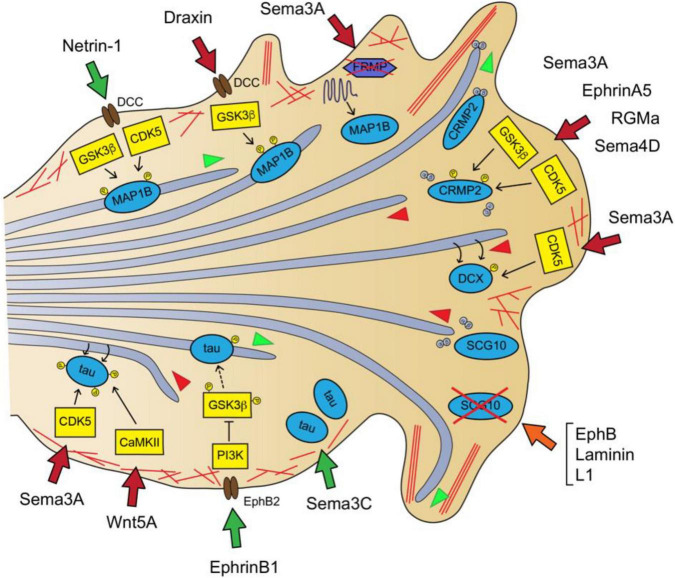
Guidance signaling downstream pathways involved in MT dynamics in the axon and GC I: MT-stabilizing, MT-destabilizing and MT-polymerization supporters. Netrin-1-DCC signaling produces MT stabilization via MAP1B phosphorylation through GSK3 and CDK5 activity (Del Río et al., 2004). Draxin binds DCC receptor and leads to MAP1B phosphorylation via GSK3β (Meli et al., 2015). Sema3A stimulates MAP1B mRNA local translation by promoting the proteasome-dependent degradation of the repressor FRMP (Takabatake et al., 2020). Sema3A, EphrinA5, RGMa or Sema4D inhibit MT polymerization by increasing CRMP2 phosphorylation via GSK3β and CDK5 (Arimura et al., 2005; Cole et al., 2006; Ito et al., 2006; Wang et al., 2013). Sema3A promotes MT destabilization by promoting DCX-fall off the MT lattice via CDK5-dependent phosphorylation of DCX (Bott et al., 2020). The combined action of EphB, laminin and L1 leads to MT overgrowth and buckling by reducing SCG10 protein levels (Suh, 2004). Sema3C increases tau protein levels (Moreno-Flores et al., 2004). EphrinB1-EphB2 signaling reduces tau hyperphosphorylation via PI3K-dependent inhibition of GSK3 (Jiang et al., 2015). Wnt5a promotes MT redistribution by stimulating CaMKII-dependent phosphorylation of tau at Ser262 (Li et al., 2014). Sema3A transiently increases tau phosphorylation at Ser202 and Thr205 via CDK5-dependent phosphorylation (Sasaki et al., 2002). MTs are shown as light purple tubes, F-actin as red lines. MAPs are represented in blue, kinases in yellow and MAP-interacting proteins in purple. Guidance cue receptors are in brown. Guidance-evoked responses are represented in green (attraction), red (repulsion) and orange (pause) arrows. MT advance and retraction are represented with green and red arrowheads, respectively.

Microtubules are heavily stabilized in the axonal shaft, whereas in the GC they are very dynamic. The stability status and polymerization rate of MTs in the axons rely on the activity of specific MAPs, such as MAP1B, tau or CRMP2, whose activities are directly regulated by axon guidance signaling pathways (Figure 2). MAP1B is a MT-stabilizing protein that associates with the lattice of dynamic MTs in the most distal region of the axon and in the GC. Studies of asymmetric laser inactivation in GCs together with genetic analyses revealed that the phosphorylated form of MAP1B is a direct effector of axon turning because selectively stabilizes MTs at the GC periphery (Black et al., 1994; Mack et al., 2000; Bouquet et al., 2004). MAP1B phosphorylation levels are increased in cortical neurons after Netrin1 treatment via GSK3β and CDK5 kinase activity. Consistently, growing axons from MAP1B-deficient CNS explants are irresponsive to netrin-1-induced chemoattraction. MAP1B mutant mice are viable but exhibit misguided cortical, thalamocortical and hippocampal axons (Table 1) (Meixner et al., 2000; Del Río et al., 2004). These dramatic axon wiring defects suggest that MAP1B is involved in additional axon guidance pathways, other than netrin-1. Indeed, the repulsive axonal guidance responses evoked by Draxin and Sema3A treatments also involve MAP1B in their downstream pathways. Draxin, which is an essential guidance cue for the development of forebrain commissural tracts, interacts with the netrin receptor DCC and activates the GSK3β-MAP1B pathway in order to induce a repulsive response in cortical axons (Meli et al., 2015). On the other hand, Sema3A treatment of hippocampal neurons increases MAP1B levels in distal axons in a local translation-dependent manner (Campbell and Holt, 2001; Li C. et al., 2009). Specifically, Sema3A induces the local degradation of the translational suppressor FMRP via the ubiquitin-proteasome pathway, which results in the increase of MAP1B mRNA-coding translation in the GC (Takabatake et al., 2020). Thus, it appears that MAP1B is a downstream mediator of both attractive and repulsive guidance cues. This high degree of MAP1B tunability could be entailed by its multiple phosphorylation sites (Kawasaki et al., 2018), sensitive to CDK5 and GSK3β activity, but further work is needed to understand the molecular mechanisms whereby MAP1B promotes GC steering.

Tau is another phospho-MAP that binds the MT lattice and stabilizes the MTs in the axon shaft, playing a critical role in axon specification, growth and branching. Tau hyperphosphorylation generally correlates with impaired MT binding and axonal MT cytoskeleton disruption (Cleveland et al., 1977; Binder et al., 1985; Drubin and Kirschner, 1986; Grundke-Iqbal et al., 1986). Similar to MAP1B, Tau is a downstream target of GSK3β and CDK5 - among other kinases (Guo et al., 2017). In particular, it was found that Sema3A treatment transiently increases phospho-tau levels in the GC of chick neuron cultures via CDK5-dependent phosphorylation previously to GC collapse (Sasaki et al., 2002). Also, Wnt5a promotes the reorganization of MTs in the GC of cortical neurons through CaMKII-dependent phosphorylation of tau within its MT-binding site (Ser262) in order to evoke a repulsive axonal response (Li et al., 2014). In opposition to this, the activation of EphB2 receptor by ephrin B1 reduces tau hyperphosphorylation through GSK3β inhibition *in vivo* in the CA3 hippocampal region of tau transgenic mice (Jiang et al., 2015). Moreover, Sema3C addition upregulates the total tau protein levels in cultured cerebellar granule neurons, preserving survival and stimulating neuritogenesis (Moreno-Flores et al., 2004). Interestingly, it was uncovered that tau promotes the co-alignment of MT and actin fibers *in vitro*, and stimulates the coordinated polymerization of both cytoskeleton networks (Elie et al., 2015). In line with this, it was recently reported that tau does not only decorates the lattice of stabilized MTs along the axon shaft but also associates to dynamic MTs aligned with actin filaments in the GC periphery of cortical neurons. Tau downregulation disrupted the MT bundling in the GC central domain, prevented MT invasion into the periphery and misoriented MT trajectories. Overall, tau loss-of-function inhibited the turning of cortical axons exposed to Wnt5a gradients (Biswas and Kalil, 2018).

The collapsin response mediator proteins (CRMPs) family are cytosolic phospho-MAPs that play important roles in the developing nervous system, including axon guidance (Nakamura et al., 2020). CRMP family name was given because its first member identified, CRMP2, was a molecular mediator of GC collapse upon stimulation with Sema3A (originally known as Collapsin) (Goshima et al., 1995). There are five human CRMPs (CRMP1-5), displaying different subcellular localization and cytoskeletal targets. Among them, CRMP2 localizes to the axon and the C-domain of the GC and controls MT polymerization/stability. Indeed, it has been observed that CRMP2 participates in axon specification, elongation, branching and guidance effect by several guidance cues (Inagaki et al., 2001; Lin et al., 2011; Higurashi et al., 2012; Yamashita and Goshima, 2012). When CRMP2 monomers are non-phosphorylated, they bind tubulin heterodimers and the complex is transported to the distal part of growing axons, by kinesin-1-dependent motor forces, to support MT polymerization and axon growth. Upon Sema3A stimulation, CRMP2 is sequentially phosphorylated at its C-terminal domain by CDK5 and GSK3β kinases, hampering its tubulin-binding properties and leading to GC collapse via MT destabilization (Figure 2). The Sema3A-induced CRMP2 inactivation is achieved by phosphorylation at Ser522 by CDK5, followed by GSK3β-dependent phosphorylation at Ser518, Thr514 and Thr509 (Fukata et al., 2002; Kimura et al., 2005; Cole et al., 2006). In addition to Sema3A, other repulsive guidance cues induced CRMP2 phosphorylation via GSK3 and/or Rho kinase to achieve GC collapse, these include Sema4D, RGMa and ephrinA5 (Arimura et al., 2005; Ito et al., 2006; Wang et al., 2013). Consistently, CRMP2 has been demonstrated to be essential for axon navigation *in vivo* because *CRMP2KO mice* exhibit axon guidance defects in peripheral nerves and in the corpus callosum (Ziak et al., 2020).

Mutations in the genes encoding the MAP doublecortin (DCX) account for the majority of the human cases of double cortex syndrome, which exhibits severe brain cortex malformations primarily attributable to neuronal migration and proliferation deficits (Gleeson et al., 1998; Bahi-Buisson et al., 2013). DCX is a MT-stabilizing phospho-protein abundant in the axonal GCs, which decorates the lattice of MTs invading the F-actin rich peripheral region of the GC (Moores et al., 2004; Tint et al., 2009). Interestingly, the double genetic deletion of DCX and its closest homolog protein doublecortin-like kinase1 (DCLK1) in mice led to widespread defects in axon tracts, affecting the corpus callosum, anterior commissure, subcortical fiber tracts and internal capsule. More specifically, the DCX mutant axons exhibit impaired transport, growth and are irresponsive to netrin-1-evoked chemoattraction, although the latter was suggested to stem from DCX regulatory effects on actin configurations (Deuel et al., 2006; Koizumi et al., 2006; Fu et al., 2013). This data suggests that DCX is required for guidance signaling-evoked axonal steering during nervous system development. Indeed, a recent study uncovered that DCX mediates the repulsive response of GCs upon Sema3A treatment (Bott et al., 2020). Bott et al. (2020) showed that DCX forms a complex with Nestin that enables DCX phosphorylation by CDK5/p35 downstream Sema3A signaling. They also demonstrated that DCX phosphorylation by CDK5 decreased its MT affinity and resulted in MT destabilization.

### Microtubule-Destabilizing, Severing and Polymerization-Inhibitory Microtubule-Associated Proteins

In addition to MT polymerization and stability, MT depolymerization and severing are also critical mechanisms for the arrangement of MT networks. Several of these MAPs have been involved in the transduction of axon guidance signaling. SCG10 (superior cervical ganglion-10)/Stathmin-2 is a neuron-specific member of the MT-destabilizing protein family of the stathmins. Stathmins bind tubulin dimers, sequestering them from growing plus-ends and thereby, promoting MT depolymerization (Charbaut et al., 2001; Grenningloh et al., 2004). SCG10 is considered an axon survival protein, highly enriched in the GCs C-domain of developing neurons, and its levels are dynamically regulated by local degradation and KIF1B-dependent axonal transport toward the GC (Shin et al., 2012; Drerup et al., 2016). Axon extension during neuron differentiation requires SGC10 activity, since its downregulation produces MT overstabilization and looping in the GC of hippocampal neurons (Morii et al., 2006). The repulsive protein EphB typically triggers GC collapse, but in the presence of laminin and L1 leads to paused GCs, which retain their normal filopodial dynamics and actin distribution. It was found that this guidance cue combination specifically reduced SCG10 levels in GC, which stimulated the invasion of long curved MTs into the GC periphery and led to GC pause (Figure 2) (Suh, 2004). Additionally, SCG10 interacts with the small RhoGTPase Rnd1, and this interaction enhances SCG10 MT destabilizing activity in neurons. Rnd1 is known to mediate the GC collapse induced by Sema4D-Plexin-B1 signaling in hippocampal neurons (Oinuma et al., 2004; Li Y.-H. et al., 2009), suggesting that SCG10 may also function downstream of the Sema4D signaling pathway.

On the other hand, the MT-severing enzymes cut MT fibers into shorter fragments, creating new local MT seeds and influencing axon branching (Sharp and Ross, 2012). Spastin is a MT-severing protein required for axon morphogenesis, associated to a degenerative disease of the corticospinal axon tracts, named Hereditary spastic paraplegia (Roll-Mecak and Vale, 2008). Recently, the alternative translation of spastin mRNA transcripts has been found to influence both motor neuron axon guidance and migration downstream of bone morphogenic protein (BMP) and neuropilin-1 signaling during zebrafish development (Jardin et al., 2018). Fidgetin-like-1 (Fignl1) is another MT-severing protein enriched in the growth cone of zebrafish growing axons, whose downregulation led to pathfinding defects in spinal motor axons and impaired larvae locomotion (Fassier et al., 2018), although no specific guidance proteins controlling Fignl1 activity have been identified.

Concerning MT polymerization inhibitors, the kinesin-4 family members KIF21A and KIF21B and the immotile kinesin-13 family member KIF2A, have been linked to neurodevelopmental malformations associated with axon growth and guidance defects in human patients (Table 1) (Yamada et al., 2003; Poirier et al., 2013; Asselin et al., 2020). More specifically, *KIF21A* is a gene risk factor for the CFEOM1 (congenital fibrosis of the extraocular muscles type-1), a developmental oculomotor nerve disorder. CFEOM1-associated *Kif21a* mutations in mice caused aberrant axon branching, stalling and misorientation defects in oculomotor nerves (Yamada et al., 2003; Cheng et al., 2014). It was reported that KIF21A decreases MT polymerization rate and suppresses MT plus-end catastrophes. KIF21A overexpression in hippocampal neurons slendered the GC morphology, stimulated axon growth and suppressed the repulsive axonal response to Sema3F (van der Vaart et al., 2013). In turn, KIF2A has been proposed to regulate axon pruning by preventing MT overstabilization in the GC. It was found that Kif2a^–/–^ mice exhibit an aberrant axonal overextension in hippocampal neurons, due to reduced MT depolymerization in the GCs (Homma et al., 2003; Maor-Nof et al., 2013).

### Microtubule-Tracking Motor Proteins

As aforementioned, dynein-driven motor forces facilitate the entry of MTs into the GC periphery, influencing neurite initiation, axon outgrowth and steering (Dehmelt et al., 2006; Myers et al., 2006; Grabham et al., 2007). In support of dynein’s role in guidance-evoked GC movements, dynein loss-of-function experiments using RNAi or Cilibrevin D revealed an impairment in NGF-evoked filopodia formation and in GC turning over substrate boundaries. However, both dynein-driven MT-sliding into the GC periphery or MT-based retrograde transport of signaling endosomes could contribute to these instructed axon movements (Myers et al., 2006; Sainath and Gallo, 2015). Likewise, MT-based kinesin-dependent anterograde transport is necessary for axonal extension and steering. For instance, the MT-sliding activity of kinesin-5 – also called Eg5 or kif11 – inhibits the MT invasion into the GC periphery and it is required for GC turning in response to repulsive substrate boundaries. It was found that an asymmetric accumulation in the GC of the phosphorylated form of kinesin-5 precedes turning, and its acute inactivation in one side of the GC elicits the MT invasion into the hampered side and GC turning (Nadar et al., 2008, 2012).

A recent study has pinpointed the kinesin KIF13B as the molecular motor responsible of Myo X localization to axons upon netrin-1 stimulation. Myo X is an actin-based motor protein that transports lipids and transmembrane receptors, such as DCC, to the filopodia tip during axon pathfinding. It was found that netrin-1 signaling increases Myo X-KIF13B interaction and its anterograde MT-dependent transport along the axons, in order to stimulate axon initiation and axon branching in the cortical commissural projections (Yu et al., 2020). The kinesin family member 1 binding protein (KIF1BP) is also necessary for a proper development of the anterior commissures and the sympathetic innervation of the gut (Hirst et al., 2017). Mutations in the *Kif1*β gene, associated to the Charcot-Marie-Tooth peripheral neuropathy, have been found to prevent KIF1Bβ binding to the insulin-like growth factor 1 (IGF1) receptor IGF1R, involved in sensory axon guidance. These mutations blocked the MT-dependent axonal transport of IGF1R and inhibited IGF1-evoked axon outgrowth (Scolnick et al., 2008; Xu et al., 2018).

The kinesin-1 motor complex has also been suggested to participate in the netrin-1-evoked repulsive response in invertebrate motor axons and is a phosphorylation target of GSK3β, a major transduction hub of various guidance signaling pathways (Teulière et al., 2011; Banerjee et al., 2018). Furthermore, mutations in gene encoding the subunit KIF5C of the kinesin-1 complex (encoded by the *Kif5* genes) have been linked to an abnormal development of the axon tracts of the corpus callosum and the internal capsule (Table 1) (Poirier et al., 2013; Michels et al., 2017). The recent analysis of a mutant mice lacking the kinesin-1 light chain KLC1 has revealed hypoplasia of the internal capsule tract, that includes corticofugal and thalamocortical axons. The innervation defects were found to be caused by an impaired kinesin-1-dependent axonal transport of the cannabinoid type-1 receptors (CB1R), and the subsequent axon unresponsiveness to endocannabinoids signaling (Saez et al., 2020).

### Microtubule Plus-Tip Interacting Proteins (+TIPs)

**FIGURE 3:**
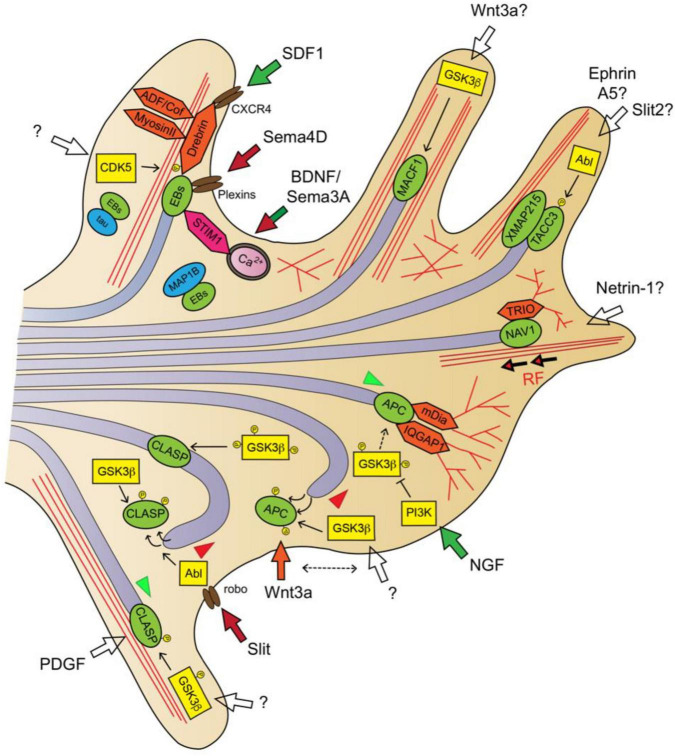
Guidance signaling downstream pathways involved in MT dynamics in the axon and GC II: +TIPs. SDF1/CXCR4 signaling activates the EB1/Drebrin module for MT remodeling (Shan et al., 2021). Sema4D/plexin signaling inhibits EB3-labeled MT polymerization (Laht et al., 2012, 2014). BDNF and Sema3A promote asymmetric MT invasion via STIM1-EB3 interaction (Pavez et al., 2019). NGF stimulates APC-dependent MT plus-end stabilization via local inhibition of GSK3β activity (Zhou et al., 2004). Wnt3a alters MT polymerization direction by misslocating APC from the MT plus-ends (Purro et al., 2008). Slit/Robo signaling promotes MT growth arrest by dissociating CLASP from the MT plus-end via Abl-dependent CLASP phosphorylation (Lee et al., 2004). High GSK3 kinase activity (poorly phosphorylated) dissociates CLASP from plus-ends, low GSK3 activity (highly phosphorylated) misslocates CLASP from plus-ends to the MT lattice, moderate GSK3 activity allows CLASP plus-end binding, MT stabilization and growth (Hur et al., 2011a). MTs are shown as light purple tubes, F-actin as red lines. +TIPs are represented in green, kinases in yellow, actin-interacting/regulatory proteins in orange and other +TIP-interacting proteins in pink. Guidance cue receptors are in brown. Guidance-evoked responses are represented in green (attraction), red (repulsion) or orange (pause) arrows. Empty arrows were used when downstream transduction pathways are unclear or guidance cues unknown. MT advance and retraction are represented with green and red arrowheads, respectively.

Plus-end tracking proteins (+TIPs) regulate MT plus-end polymerization and stability, and mediate interactions between the MT ends and actin fibers, organelles and plasma membrane (van de Willige et al., 2016). Evidences obtained during the last 15 years have demonstrated that axon guidance signaling pathways directly target via regulation of +TIPs’ activity and localization (Figure 3) (Bearce et al., 2015; Cammarata et al., 2016; Voelzmann et al., 2016).

Microtubule end-binding (EB) proteins are the most abundant +TIPs in cells. EBs (EB1, EB2, and EB3) directly associate with MT plus-ends through their N-terminal calponin homology (CH) domain and are autonomous regulators of plus-end dynamics. MT tip-tracking of EBs mainly correlates with MT polymerization episodes, since it favors a continuous polymerization and reduces the number of catastrophes. Importantly, EBs and are also scaffold-providers for other +TIPs through their C-terminal domain, and for this reason EBs are considered master regulators of +TIP network. EB1 protein is ubiquitous, whereas EB3 is predominantly expressed by neurons, and both EB1 and EB3 are necessary for axon extension (Akhmanova and Steinmetz, 2015; van de Willige et al., 2016). Semaphorin4D can influence the EB3-labeled MT plus-ends polymerization dynamics in hippocampal neurons, and it was found that both EB1 and EB3 interact with the intracellular domains of the Plexin-A2, Plexin-B1, and Plexin-B3 Semaphorin family receptors (Laht et al., 2012, 2014). This data suggest that the semaphorin-plexin-EB pathway may regulate MT dynamics during axon pathfinding. When MT plus-ends enter the actin-rich P-domain of GCs, EB3 recruits the F-actin-binding protein Drebrin to couple growing MT tips to actin filaments. Hence, the EB3-Drebrin module facilitates the invasion of exploratory MTs into the GC periphery, and enables growth cone formation and neuritic elongation (Geraldo et al., 2008). It has been recently proposed that the EB1-Drebrin module at plus-ends interacts with the chemokine receptor type 4 (CXCR4) upon stromal cell-derived factor-1 (SDF-1) signaling. The chemokine SDF-1 can regulate axonal elongation and branching, and the SDF-1/CXCR4/Drebrin/EB1 pathway appears to be critical for SDF-1-induced MT cytoskeleton remodeling during neuronal motility (Pujol et al., 2005; Shan et al., 2021). Furthermore, EB3 colocalizes at the MT plus-ends within the GC filopodia with the stromal interacting molecule (STIM1), which is a calcium-sensing protein that mediates GC steering in response to various axon guidance cues (Mitchell et al., 2012; Pavez et al., 2019). New data revealed that EB3-STIM1 interaction at MT plus-ends is calcium-sensitive and STIM1 instructs the asymmetric invasion of EB3-labeled MT plus-end into the motile GC side downstream of BDNF or Sema3A signaling in sensory neuron cultures. Moreover, *in vivo* experiments in zebrafish showed that the EB3-STIM pathway regulates the axon guidance of spinal motor neurons (Pavez et al., 2019). In addition, EB1/3 proteins can bind MAP1B and Tau, and this interaction sequesters EBs from MT plus-ends and jeopardizes MT growth (Tortosa et al., 2013; Sayas et al., 2015). Because MAP1B and tau are downstream effectors of several axon guidance pathways, their interaction with EBs could indirectly influence the MT dynamics and the assembly of the +TIP complex on account of its dependence on EB scaffold.

One of the pioneer studies that assigned + TIPs a prominent role in axon guidance refers to the cytoplasmic linker associated protein (CLASP) and the Slit-evoked repellent response (Lee et al., 2004). CLASP decorates the MT plus-ends that polymerize over F-actin bundles in the GC periphery, and its overexpression causes MT overstabilization, looping and prevents their extension beyond the transition zone (Hur et al., 2011a). Orbit/MAST, the CLASP ortholog in invertebrates, is an Abelson tyrosine kinase (Abl) target downstream of Slit/Robo signaling that mediates repulsion. These evidences served the authors to propose that focal Slit stimulation in one side of the GC provokes asymmetric activation of the Abl-CLASP pathway and MT growth arrest, entailing a GC movement away of the source of Slit (Lee et al., 2004). CLASP is recruited to MT plus-ends through EB binding, although it contains tumor overexpressed gene (TOG) domains which can serve as tubulin-binding modules (Mimori-Kiyosue et al., 2005; Al-Bassam and Chang, 2011). Indeed, it has been observed that CLASP localization in the MTs can alternate between the plus-end and the MT lattice, based on its phosphorylation by GSK3β. These MT-binding activities determine the degree of MT protrusion and subsequent axon growth in an opposing manner. A high GSK3 kinase activity promotes CLASP dissociation from MT plus-ends, leading to MT destabilization and impaired axon growth, while a moderate GSK3 activity allows CLASP plus-end binding, promoting MT stabilization and axon extension. A low GSK3 activity leads to CLASP localization to the MT lattice, producing MT overstabilization and looping in the GCs, and axon growth attenuation (Akhmanova et al., 2001; Hur et al., 2011a). Given that GSK3 kinase activity is fine-tuned by many downstream axon guidance pathways, CLASP may also act as transducing factor of other extracellular guidance cues (Hur and Zhou, 2010).

APC (Adenomatous Polyposis Coli Protein) is a critical tumor suppressor, initially reported as Wnt-signaling regulator. In the Wnt pathway, APC forms a complex with GSK3 and other proteins to target and degrade the oncoprotein β-catenin (Stamos and Weis, 2013). In addition to this function, APC is an EB-binding +TIP that stabilizes the MT plus-ends and, similar to CLASP, this activity is abolished by GSK3β-mediated phosphorylation (Nakamura et al., 2001; Zumbrunn et al., 2001). In neurons, APC is transported toward the distal region of the growing axon by kinesin-1 motor forces, and distributes asymmetrically within the GC. Indeed, the local accumulation of APC in one side of the GC anticipates the steering movement of the axon in this axial direction (Koester et al., 2007; Ruane et al., 2016). It was demonstrated that the focal stimulation of GCs with Nerve Growth Factor (NGF) produces the localized inactivation of GSK3β via PI3K activity, which enables APC-dependent stabilization of MT plus-ends in GCs and rapid axon elongation (Zhou et al., 2004). Additionally, the treatment with the GC-pausing guidance cue Wnt3a led to altered MT growth directionality in the GC by misallocating APC from the MT plus-ends at the P-domain (Purro et al., 2008). *In vivo*, despite initial contradictory results obtained in *Drosophila*, APC has been shown to play an important role in neural circuits formation. APC mutant mice exhibit gross misrouting defects in the internal capsule, posterior commissure or thalamocortical axons, and APC-deficient neurons displayed an abnormal axonal arborization and curling at the tips (Rusan et al., 2008; Yokota et al., 2009; Jin et al., 2018). Besides its MT-stabilizing role at the plus-end, APC participates in the MT-based transport of mRNAs, such as those encoding β-actin, Tubb2b or the dynein complex subunit Lis1, toward the axon. Importantly, APC association with their mRNA targets to transport them along sensory axons is triggered by exogenous stimulation with NGF (Preitner et al., 2014; Villarin et al., 2016; Baumann et al., 2020).

APC2, APC’s brain specific homolog, is a MT-binding protein and contains a C-terminal region with MT tip-tracking properties. APC2 localizes to GCs of chick retinal axons and participates in retinotectal axon guidance through regulation of MT stability. *Apc2*-knockdown display an attenuated response to ephrin-A2 in retinal ganglion cells (Shintani et al., 2009; Kahn et al., 2018). Also in retinal neurons, APC2 has been identified as a direct target of the transcription factor Zic2, the main determinant of axon midline avoidance, which also regulates the guidance receptors EphB1 and Unc5c (Herrera et al., 2003, 2019b; Escalante et al., 2013; Kridsada et al., 2018; Murcia-Belmonte et al., 2019). In ipsilaterally projecting neurons, *Apc2* expression is intrinsically downregulated by Zic2 likely to facilitate Wnt5a and ephrinB2-mediated axon repulsion at the optic chiasm (Morenilla-Palao et al., 2020).

Microtubule-actin crosslinking factor 1 (MACF1), also known as actin-crosslinking factor 7 (ACF7), is a large multidomain protein of the spectraplakin family, highly expressed in the nervous system. MACF1 interacts with MT plus-ends and enables MT capture by F-actin, facilitating MT polymerization over F-actin bundles at the cellular periphery (Kodama et al., 2003; Wu et al., 2008). MACF1 can directly interact with MTs through its C-terminal Gas2-related (GAR) domain or indirectly by EB binding, and simultaneously binds F-actin through its N-terminal calponin-homology (CH) domains. In addition, MACF1 has a C-terminal AAA-ATPase domain that can exert molecular forces over the MT cytoskeleton (Moffat et al., 2017). Genetic studies in *Drosophila* showed that MACF1 homolog protein *Shot* is required for axon extension and midline guidance, and that its MT plus-tip tracking enabled by EB1-binding is necessary to maintain an organized MT network in axons (Lee et al., 2007; Alves-Silva et al., 2012). Consistently, mammalian MACF1 also regulates neuronal MTs configurations and filopodia formation, a role dependent on both MACF1 F-actin- and MT-binding domains (Sanchez-Soriano et al., 2009). MACF1 mediates Wnt/GSK3β signaling, and its loss-of-function in mice phenocopied the early developmental defects observed in Wnt3^–/–^ embryos. Specifically, the conditional deletion of MACF1 in neural progenitors produced the agenesis of the anterior commissure and an abnormal development of the thalamocortical fibers and the hippocampal commissure in neonatal mice (Chen et al., 2006; Goryunov et al., 2010). Moreover, MACF1 downregulation in cortical early postmitotic neurons interfered with the normal arrangement of MTs and F-actin networks in neurites, inhibited neuron radial migration and disrupted callosal axon innervation (Ka et al., 2014; Ka and Kim, 2016). Interestingly, heterozygous missense mutations in the MT-binding GAR domain of MACF1 have been recently identified in human individuals exhibiting axonal midline crossing phenotypes, among other defects (Table 1) (Dobyns et al., 2018).

Neuron navigator-1 (NAV1) belongs to the +TIP family of Navigators (NAVs), which is represented by NAV1, NAV2, and NAV3 in mammals. NAVs are large proteins, carrying N-terminal calponin-homology (CH) domains and an intriguing C-terminal ATPase domain, which have been associated to axon outgrowth (Martínez-López et al., 2005; van Haren et al., 2009; McNeill et al., 2010; Abe et al., 2014). In particular, NAV1 expression was found to be largely restricted to the developing nervous system being enriched in the neuritic tips and GCs. Hindbrain neurons lacking NAV1 do not respond to Netrin-1, which suggested a function downstream of Netrin-1 signaling (Martínez-López et al., 2005; van Haren et al., 2014). It was recently described that, similar to CLASP or MACF1, NAV1 is an EB-dependent +TIP that can directly bind actin fibers *in vitro*, and data suggest that it crosslinks MT plus-ends to the F-actin network within the GCs from mammalian cortical neurons (Sánchez-Huertas et al., 2020). In the proposed model, EB proteins recruit NAV1 to the MT tip during polymerization inside F-actin-enriched regions. Following EB-complex disassembly and MT growth arrest, NAV1 switches to an EB-independent form of association with the MT plus-end and stabilizes it, reducing the frequency of MT shrinkage. Thereafter, paused plus-ends undergo retrograde translocation coupled to F-actin retrograde flow via MT-NAV1-F-actin crosslinking (Sánchez-Huertas et al., 2020). However, NAV1 sequence does not possess a CH domain for actin binding, neither GAR nor TOG domains for direct MT interaction. Hence, the specific NAV1 domains responsible for direct F-actin binding and whether NAV1-MT interaction requires an intermediary autonomous MT-binding protein, still remain to be elucidated. NAV1 was also found to mediate the chemoattractive response of cortical axons toward a source of netrin-1 and the radial migration of pyramidal neurons during *in vivo* corticogenesis (Sánchez-Huertas et al., 2020). NAV1 mRNA and protein levels are highly enriched in developing cortical layer V, mainly populated by projection neurons innervating subcortical targets, such as the brainstem or the spinal cord (Martínez-López et al., 2005; Sorensen et al., 2015). This observation suggests that NAV1 might be required for axonal navigation by layer V projection neurons in particular, and allows to hypothesize that *ad hoc* neuron cytoskeletal machinery may transduce guidance signaling differently in specific neuron subtypes.

Recent evidences suggest that the module formed by the +TIPs XMAP215 (chTOG or CKAP5 in mammalian cells) and transforming acidic coiled-coil 3 (TACC3) protein represent an unconventional EB-independent regulatory mechanism of MT plus-end dynamics downstream axon guidance signaling. XMAP215 is a conserved processive MT polymerase that catalyzes tubulin addition into the polymer while it tracks the MT plus-ends (Gard and Kirschner, 1987; Brouhard et al., 2008). Although XMAP215 and EB1 can act synergistically to promote MT growth, XMAP215 does not require EB proteins to track MT plus-ends because it binds MTs directly through its five N-terminal TOG domains. Indeed, XMAP215 locates to the extreme MT plus-end several tens of nanometers ahead of the region bound by EB1 and remains attached to the MT plus-end even during shrinkage events (Nakamura et al., 2012; Zanic et al., 2013; Maurer et al., 2014). XMAP215 downregulation greatly increases MT catastrophe frequency throughout the neuron cell body and compromises hippocampal axon growth (van der Vaart et al., 2012). While in most cellular contexts XMAP215 downregulation decreases MT plus-end growth, in GCs it accelerates MT plus-end velocities. This increase was proposed to arise from higher MT anterograde translocation rates in the GCs, likely due to the uncoupling between MT plus-ends and the F-actin retrograde flow in the absence of XMAP215 (Lowery et al., 2013). More recently, it was reported that XMAP215 directly binds actin fibers and it is necessary for MT-F-actin alignment in the GCs. Indeed, it has been demonstrated that XMAP215 regulates MT invasion into GC filopodia, influences GC morphology and protrusion, and mediates the repulsive response to ephrinA5 (Slater et al., 2019).

TACC3, first identified as a regulator of astral and spindle MT length, has been classified as + TIP on account of its binding to MT plus-ends through its TACC domain and assigned a role in plus-end dynamics and axon outgrowth (Gergely et al., 2000; Nwagbara et al., 2014). TACC3 interacts with XMAP215 in the distal region of MT plus-ends, and they are important for each other’s localization to the plus-end. Indeed, TACC3 and XMAP215 can rescue each other’s downregulation phenotypes in axon elongation, and it has been suggested that TACC3 strengthens the XMAP215-TACC3 complex binding to MTs in order to drive polymerization activity (Nwagbara et al., 2014; Erdogan et al., 2017). TACC3 is a phosphorylation target of the kinase Abl, whose activity is known to be regulated by axon guidance signaling (Kannan and Giniger, 2017). A TACC3 phospho-null mutant failed to localize at MT plus-ends in GCs, leading to an increase of MT invasion into the filopodia and impaired axon pathfinding. Interestingly, the overexpression of TACC3 interfered with the responsiveness of axons from *Xenopus* neurons explants upon Slit2 and Ephrin-A5 signaling (Erdogan et al., 2017, 2020).

## Microtubules Instruct F-Actin Remodeling in the Growth Cone

The interaction of MTs with actin filaments and the involvement of MAPs in this crosstalk is a matter of study since more than 40 years (Griffith and Pollard, 1978; Selden and Pollard, 1983). This body of work has established that axonal navigation responses to guidance signals demand an intense and coordinated cytoskeleton remodeling, during which both MT and F-actin influence each other’s dynamics. As aforementioned, F-actin dynamics influence MT advance and retrograde translocation in the GC periphery (Schaefer et al., 2002; Zhou et al., 2002). Even along the axonal shaft, F-actin structures contribute to the maintenance and dynamics of the MT networks (Winans et al., 2016; Qu et al., 2017). Conversely, the entry of MT plus-ends into the actin-rich cortical regions promotes changes in actin-based structures of the growth cone. Seminal works reported that drugs that inhibit MT dynamics, without appreciable depolymerization, halt the bundling and splaying movements in the peripheral GC domain. At higher concentrations, MT drugs resulted in the loss of lamellipodia and an increase in filopodial length but not filopodial number in the GCs (Tanaka et al., 1995; Gallo, 1998). MT dynamics were also found to be necessary for the maintenance of the F-actin foci that formed in GCs in response to substrate adhesions. In particular, it was found that dampening MT dynamics with drugs suppressed focal F-actin assembly upon laminin signal detection, while the washout of the drug restored these foci, indicating that extracellular signaling can influence F-actin in the GC via MTs (Grabham et al., 2003; Suter et al., 2004). More recently, live microscopy experiments on hippocampal cultures exposed to MT-targeting drugs, revealed that decreasing MT stability significantly reduced F-actin treadmilling in the GC periphery of the nascent axons. Conversely, increasing the MT stability or the MT density in axons resulted in an increase in F-actin dynamics in GCs (Zhao et al., 2017). Together, this data showed that MT dynamics influence F-actin turnover in the GC periphery and revealed the critical role of MTs in the maintenance of the actin-based lamellar and filopodial structures of GCs.

The MT-stabilizing MAPs MAP1B and Tau can simultaneously bind actin filaments and contribute to MT-actin coalignment in the GC. Additionally, MAP1B and tau can stimulate F-actin polymerization and bundling (Villarroel-Campos and Gonzalez-Billault, 2014; Elie et al., 2015; Biswas and Kalil, 2018). However, F-actin and MTs crosstalk mainly takes place at the MT plus-ends and the most suitable candidates to assemble both networks are the +TIPs (Bearce et al., 2015; Cammarata et al., 2016). A minimal engineered version of the + TIP MACF1, containing N-terminal CH domains and C-terminal EB-binding motifs – denominated TipAct – showed efficient MT plus-end tracking and binding to F-actin structures at the cell periphery. TipAct showed low F-actin binding affinity *in vitro*, but its local concentration at MT plus-ends allowed MT tips to link actin fibers. Therefore, when TipAct was added to mixed preparations of purified tubulin and actin, it enabled MTs to transport, pull and bundle actin fibers, globally arranging F-actin configurations (Preciado López et al., 2014). The +TIP CLIP170 also exhibited capacity to stimulate *in vitro* F-actin elongation in MT-actin re-constitution experiments via CLIP170 interaction with the formin mDia1. It was shown that CLIP170-mDia1 complexes are recruited to growing MT ends by EB1 and stimulate F-actin polymerization from the MT surface. The actin fibers remained attached to MTs until they spontaneously detached or were released by a MT catastrophe event (Henty-Ridilla et al., 2016). Furthermore, a recent study performed in hippocampal neurons uncovered that MT plus-ends assemble F-actin networks in the GC periphery in an APC-dependent manner (Efimova et al., 2020). APC modulates the activity of various actin regulators, such as the formin mDia or IQGAP1, which is a downstream effector of Rac1 and Cdc42 GTPases (Watanabe et al., 2009; Okada et al., 2010). In support of this data, electron microscopy analysis reported that APC targets MT plus-ends at the MT-actin interphase in the GC periphery of hippocampal neurons, and that APC is necessary for the local assembly of branched actin filaments in these GCs and also for filopodial protrusions. Importantly, encounters of dynamics APC-positive MT tips with the membranous cell cortex induced local actin-rich protrusions (Efimova et al., 2020). These experiments demonstrate that MTs are important regulators of actin configurations in the GC, either by controlling F-actin treadmilling and polymerization, or by templating F-actin organization.

Other +TIPs have also been shown to bind actin fibers *in vitro* and/or influence F-actin configurations in the GC or filopodial dynamics. CLASP directly binds F-actin *in vitro* and its downregulation alters the F-actin networks in the GC of invertebrate neurons. It was described that CLASP-depleted GCs lack a dense F-actin meshwork and contain less actin bundles, and that lamellipodial architecture relies on CLASP interaction with MTs. Interestingly, CLASP binding to both MTs and F-actin was found to be regulated by Abl-dependent phosphorylation upon serum or platelet-derived growth factor (PDGF) signaling (Marx et al., 2013; Engel et al., 2014). Growing MT plus-ends that enter F-actin-rich areas of the GC are decorated with EB1-NAV1 complexes, and NAV1 transiently crosslinks MTs to F-actin. It has been shown that NAV1 restrains filopodial dynamics and compacts the GC morphology, suggesting a role in F-actin remodeling perhaps through recruiting the RhoGEF Trio to MT plus-ends invading the GC periphery. In addition, NAV1 protein mediates the netrin-1-evoked chemoattraction over cortical axons (van Haren et al., 2014; Sánchez-Huertas et al., 2020). Similarly, the EB3-Drebrin module also contributes to MT-actin coordination and moreover, drebrin inhibits myosin II activity, reduces cofilin-induced severing of F-actin and stabilizes F-actin (Geraldo et al., 2008; Mizui et al., 2009; Mikati et al., 2013; Grintsevich and Reisler, 2014; Zhao et al., 2017). Drebrin’s F-actin bundle-binding activity is controlled via CDK5 phosphorylation, and CDK5 is a molecular hub downstream various guidance signaling pathways (Gordon-Weeks, 2017). Yet, the specific guidance cues leading to Debrin’s phosphorylation via CDK5 remain to be identified. In addition, the protein Growth arrest-specific 2-like 1 (Gas2L1) has a domain composition similar to MACF1 and a recent study revealed that it performs as a MT-F-actin cytolinker. The simultaneous interaction of Gas2L1 with MTs and actin fibers *in vitro* released its autoinhibition. Thus, it was proposed that MT-F-actin crosslinking via Gas2L1 in actin-rich regions promotes local F-actin stabilization and influences axon outgrowth and branching. In contrast, MT dynamics were unaffected in neurons following Gas2L1 depletion (Willige et al., 2019).

Other emerging players of MT-actin crosstalk in the GC of navigating axons are the formins, a protein family composed by F-actin assembly factors. Formins may also display MT stabilizing and organizing activities, in some cases independently of their actin polymerization roles, to regulate axon pathfinding (Kawabata Galbraith and Kengaku, 2019). For instance, mDia1 and mDia3 appear to mediate the axonal response to ephrinA5, ephrinB3, Sema3A or SDF1-α in different neuron types, and knockout mice models demonstrate that they are required for spinal cord midline crossing (Arakawa et al., 2003; Thurston et al., 2012; Toyoda et al., 2013). In *Drosophila*, Disheveled-associated activator in morphogenesis (DAAM) is a downstream effector of Wnt5 signaling that exhibits MT-F-actin crosslinking activity during axonal development. It has been proposed that DAAM reshapes filopodia and actin structures in GCs via interaction with +TIPs at MT plus-ends (Gombos et al., 2015; Szikora et al., 2017). Another member of the formin family, FMN2, also participates in the stability of focal adhesions and the generation of traction forces in filopodia and facilitates MT capture by F-actin bundles in the GC of spinal neurons. Interestingly, chick FMN2-depleted spinal commissural neurons exhibited midline crossing defects (Sahasrabudhe et al., 2016; Kundu et al., 2021).

## Future Directions

During the last years, our understanding of the molecular mechanisms and proteins involved in the cytoskeletal transduction of axon guidance signaling has greatly progressed. While the list of upstream guidance cues and receptor families has not significantly grown, novel combinatorial mechanisms involved in signal transduction and cytoskeleton-regulatory proteins recipient of guidance information are continuously emerging (Stoeckli, 2018; Zang et al., 2021). Among the latter, Microtubule-Associated Proteins (MAPs) represent a significant group. Yet, the role of numerous MAPs in axon guidance is still unexplored and the intricate mechanisms of MT-F-actin coordination in the GC remain unclear.

Despite significant advances, experimental designs performed in non-neuronal cells or limited to few cytoskeleton-regulatory proteins and guidance cues, may not reflect the full scope of cytoskeletal changes triggered by extracellular guidance signaling during axon pathfinding. As a sign of the complex regulation of physiological MT dynamics in cells, recent data has demonstrated that MAP combinations exert collective effects on MTs and MAPs must follow certain hierarchies in their MT recruitment to achieve specific functions (Niu et al., 2019; Hahn et al., 2021). Besides, in addition to stereotyped mechanisms of guidance signal transduction - including regulated guidance receptor expression, dimerization or trafficking - other molecular mechanisms underlying axon guidance decisions are being characterized (Harada et al., 2020; Klein and Pasterkamp, 2021). For instance, it was recently shown that retinal ganglion cell (RGC) axons exhibit an intrinsic pathfinding program in absence of any paracrine signaling from the surrounding tissue (Harada et al., 2020). This sort of cell-autonomous guidance mechanism could act in coordination with extrinsic guidance cues to enable divergent axonal responses to the same guidance information. Indeed, mathematical models predict that extracellular signaling may instruct axon guidance by simply controlling neuron-intrinsic stochastic transitions between GC states (Padmanabhan and Goodhill, 2018).

In summary, we believe that further experiment conceptualization approaching the molecular mechanisms of axon guidance should keep in mind that: (i) downstream guidance pathways may simultaneously target both actin and MT regulatory proteins, enabling an intricate cytoskeletal crosstalk in the GC, (ii) the expanding and diverse MAP network can exert combined effects on MT dynamics, (iii) GC-intrinsic states (stalled/dynamic) and *ad hoc* cytoskeletal machinery may influence axon behavior in specific neuron subtypes, and (iv) GCs navigate a three-dimensional environment and transduction pathways described in the literature may not perfectly match with those operating in living organisms. Furthermore, the use of transcriptomics and proteomics techniques applied to the GC fraction of specific neuron subpopulations (Poulopoulos et al., 2019), high-resolution cytoskeleton imaging (Jung et al., 2020; Katrukha et al., 2021) or 3D microfluidic assays (Spijkers et al., 2021) will expand our understanding of the steered GC locomotion mechanisms and reveal new molecular specificities in the long-range growing axons accounting for neural circuits development.

## Author Contributions

CS-H wrote the article and made the figures. EH edited the article. Both authors contributed to the article and approved the submitted version.

## Conflict of Interest

The authors declare that the research was conducted in the absence of any commercial or financial relationships that could be construed as a potential conflict of interest.

## Publisher’s Note

All claims expressed in this article are solely those of the authors and do not necessarily represent those of their affiliated organizations, or those of the publisher, the editors and the reviewers. Any product that may be evaluated in this article, or claim that may be made by its manufacturer, is not guaranteed or endorsed by the publisher.
